# Time-resolved energy-share reconstruction of individual glycolytic flux kinetics reveals duration-dependent bias in elite sprint cycling

**DOI:** 10.1007/s00421-026-06397-1

**Published:** 2026-08-29

**Authors:** Katharina Dunst, Benedikt Meixner

**Affiliations:** 1 Institute for Applied Training Science (IAT), Department of Endurance Sports, Leipzig, Germany; 2 Integrative and Experimental Exercise Science & Training, Department of Sport Science, Julius-Maximilians-Universität Würzburg, Würzburg, Germany; 3 Department of Sport Science and Sport, Friedrich-Alexander-Universität Erlangen-Nürnberg, Erlangen, Germany

**Keywords:** Lactate kinetics, Energy metabolism, Sprint performance, Exercise testing, Cycling

## Abstract

**Background:**

Maximal lactate accumulation rate (vLamax) is commonly used as a surrogate of anaerobic glycolytic power. Conventional protocols calculate vLamax as a mean rate over a fixed sprint window after subtracting an alactic phase, even though glycolytic flux rises, peaks, and declines within seconds. Fixed-duration protocols may therefore underestimate the instantaneous peak rate.

**Methods:**

Seventeen elite track cyclists performed 12 s and 60 s maximal isokinetic sprints with continuous power, gas exchange, near-infrared spectroscopy, and blood lactate. Total metabolic energy (from dynamic gross efficiency), aerobic energy, and phosphocreatine energy were reconstructed, and the glycolytic residual gave vLa(t), vLamax, and time to peak.

**Results:**

The indirect assessment yielded higher vLamax than the gold standard (0.91 ± 0.18 vs. 0.82 ± 0.17 mmol/L/s; *p* < 0.001), while predicted 12 s lactate accumulation matched measured values (7.67 ± 1.77 vs. 7.58 ± 1.60 mmol/L; *p* = 0.464). Peak vLa occurred at 9.1 ± 1.4 s, before the 12 s endpoint.

**Conclusion:**

A single maximal sprint suffices to reconstruct the individual glycolytic flux trajectory (its onset, peak, and post-peak decline), and the cumulative lactate accumulation was independently validated against a separate 12 s sprint. On this basis, fixed-duration protocols appear to underestimate instantaneous peak vLamax by averaging across the post-peak decline (mean bias ~ 10%). Durations around 9–10 s may therefore offer a physiologically grounded compromise for vLamax assessment in elite cycling.

## Introduction

High-intensity exercise performance is fundamentally determined by the maximal rate at which metabolic pathways can resynthesize adenosine triphosphate to sustain muscle contraction (Heck et al. 2003). Accordingly, quantifying the relative contributions of the oxidative, alactic (ATP-PCr), and glycolytic energy systems is essential for identifying physiological limitations, guiding training interventions, and optimizing performance (Langley et al. 2025). Oxidative metabolism can be directly quantified using expired gas analysis (Van Hooren et al. 2024), and validated indirect methods exist to assess alactic energy turnover based on excess post-exercise oxygen consumption and near-infrared spectroscopy (Knuttgen 1970; McCully et al. 1994). In contrast, no established non-invasive gold standard exists for the quantification of glycolytic energy metabolism under maximal exercise conditions (Langley et al. 2025).

The maximal lactate accumulation rate (vLamax) has therefore emerged as the primary surrogate marker of glycolytic power and is defined as the peak rate of lactate accumulation in blood during maximal exercise (mmol/L/s) (Wackerhage et al. 2025). During high-intensity contractions, glycolysis rapidly generates pyruvate, which is partly oxidized in mitochondria and partly reduced to lactate and transported into the bloodstream. Although vLamax underestimates total glycolytic flux because a fraction of pyruvate is oxidized concurrently (Yang et al. 2023), it provides a practical and sensitive indicator of glycolytic capacity and is widely used in performance diagnostics for sprint- and acceleration-dominated disciplines (Quittmann et al. 2020; Dunst et al. 2026).

Classically, vLamax is calculated as the ratio of net blood lactate increase (ΔBLC) to effective exercise duration, the latter obtained after subtraction of an initial alactic phase (talac) predominantly supported by phosphocreatine breakdown (Mader 1994). Despite decades of application, substantial methodological heterogeneity persists in protocol duration (3–60 s), talac corrections, and terminology (Langley et al. 2025). A conceptual gap underlies all fixed-duration protocols: vLamax denotes the peak, muscle-level glycolytic rate, whereas the formula returns a net blood lactate accumulation rate averaged across the sampling window and uses it as a surrogate for that peak. This surrogate is adequate only if glycolytic flux remains approximately constant across the window, which is physiologically implausible because flux rises, reaches a transient peak, and then declines (Erecińska et al. 1995; Mader 2003; Gastin 2001). Modeling approaches place this peak between 4 and 12 s depending on modality, with an optimum near 9 s in cycling (Langley et al. 2025; Porter and Langley 2025), earlier than the conventional 12–15 s endpoint. Because the averaging window extends beyond the peak, the mean rate is pulled below it, the more so the longer the window. This explains why shorter protocols (~ 10 s) yield systematically higher vLamax than longer ones (Langley et al. 2024; Haase et al. 2024, 2025). Two further factors bias the absolute value: concurrent oxidative disposal of pyruvate (3–10% of turnover even in short sprints; Yang et al. 2023) means ΔBLC underestimates gross glycolytic flux, and because talac enters the denominator, a one-second deviation can alter vLamax by more than 10% in short protocols (Hauser et al. 2014; Dunst and Hesse 2023e). None of these contributions can be fully separated within a fixed-duration design.

Resolving these limitations requires knowledge of the full time course of glycolytic flux, not just its cumulative integral over an arbitrary interval. The energy-share approach (Di Prampero 2005; di Prampero and Ferretti 1999; Ferretti and Di Prampero 2026; Beneke et al. 2002) provides a mechanistically grounded framework to address these limitations by partitioning total metabolic energy turnover into phosphocreatine, glycolytic, and aerobic components. When combined with time-resolved modeling of dynamic gross efficiency, this approach enables second-by-second reconstruction of metabolic power and substrate contributions from a single maximal effort, yielding not only a peak vLamax estimate, but the complete lactate formation trajectory including its onset, time to peak, and post-peak decline. Earlier work has compared vLamax across fixed sprint durations (Langley et al. 2024; Porter and Langley 2025) or fitted a group-level optimum. Here we instead reconstruct the individual flux trajectory from a single effort, providing subject-specific values for onset, peak, and decline.

This approach is particularly feasible in sprint cycling, where high-resolution mechanical power measurement is standard practice at the elite level. The remaining inputs – breath-by-breath gas exchange, near-infrared spectroscopy, and capillary blood lactate – are common additions in laboratory-based performance diagnostics and can all be obtained from a single maximal sprint without additional testing protocols. Therefore, the primary aim of this study was to develop an indirect, time-resolved energy-share method, incorporating dynamic gross efficiency, to reconstruct the glycolytic flux trajectory, including its peak (vLamax), onset, and post-peak decline, from a single maximal sprint. Because absolute vLamax estimates from the indirect method depend on model assumptions that cannot be independently verified, we first established model validity through a criterion that is directly measurable: cumulative blood lactate accumulation. We tested two hypotheses: (1) the indirect method provides valid estimates of cumulative blood lactate accumulation during a 12 s sprint – a necessary prerequisite for interpreting the modeled kinetics – and (2) time-resolved reconstruction would localize the peak glycolytic rate in time and quantify the resulting averaging bias of fixed-duration estimates. On mechanistic grounds we expected the peak to precede the 12 s endpoint, and although the magnitude of this bias depends on the assumed phosphocreatine kinetics, its direction does not.

## Methods

### Study design and ethics

This study employed a cross-sectional laboratory design. All procedures were approved by the Ethics Committee of the Institute of Applied Training Science (ER_2022.18.03_10) and conformed to the Declaration of Helsinki. All participants provided written informed consent prior to participation. Reporting followed the STROBE guidelines for observational studies.

### Participants

Seventeen elite track cyclists (14 male, 3 female) participated voluntarily (age 20.3 ± 2.9 years; height 1.83 ± 0.06 m; body mass 84.2 ± 9.0 kg; body fat 14.2 ± 3.0%; V̇O₂peak 55.5 ± 7.7 ml/kg/min (highest value from a separate incremental test; no plateau criterion applied); cycling efficiency 13.7 ± 2.9 ml O2/min/W; maximal mean pedal force Fmax 1150 ± 173 N; optimal pedalling rate PRopt 144 ± 16 rpm; maximal power Pmax 1590 ± 411 W; phosphocreatine capacity WPCr, TOT 48.9 ± 14.1 kJ (fast EPOC component; see Model calibration). V̇O₂peak and cycling efficiency were obtained from a separate incremental exercise test. Neuromuscular characteristics (Fmax, PRopt, Pmax) were derived from individual fatigue-free force–velocity profiles based on the initial acceleration phase of each sprint (≤ 3 s) supplemented by a low-resistance sprint at pedalling rates ≥ 160 rpm (Dunst et al. 2023b). Due to the limited number of female participants (*n* = 3), data were pooled across sexes for all analyses.

Inclusion criteria were: (1) participation in national or international track cycling competitions within the preceding year; (2) high neuromuscular performance characterized by linear force-velocity profiles (R² > 0.95); and (3) absence of cardiovascular, metabolic, or musculoskeletal disease confirmed by medical screening. Participants refrained from strenuous exercise and alcohol for 24 h before testing and maintained habitual diet and hydration. An a priori sample size estimation (G*Power 3.1.9.2; α = 0.05, power = 0.95, effect size f = 0.50) indicated a minimum sample of *n* = 12.

### Experimental design

All testing was completed during a single laboratory visit to minimize biological variability. After a standardized warm-up (6 min at 1.0–1.5 W/kg, one 3 s maximal acceleration, followed by 10 min passive recovery), participants performed two maximal isokinetic sprint tests at a fixed cadence of 120 rpm, reflecting typical mean pedalling rates in international sprint competitions (Dunst et al. 2023c): a 12 s sprint and a 60 s sprint. Test order was fixed (12 s before 60 s) because the 60 s sprint induces prolonged metabolic perturbation and neuromuscular fatigue that cannot be fully reversed within practical recovery periods, whereas the 12 s sprint allows complete recovery within 30–60 min as verified by return of blood lactate and glucose to baseline concentrations. Recovery periods (30–60 min) were individualized until blood lactate and glucose concentrations returned to baseline. Participants were instructed to accelerate maximally to the target cadence and maintain maximal voluntary effort throughout. Strong verbal encouragement was provided. Following each sprint, participants remained seated for 30 min for assessment of excess post-exercise oxygen consumption (EPOC).

### Measurement procedures

All sprints were performed on an SRM cycle ergometer (Schoberer Rad Messtechnik, Jülich, Germany) equipped with a 9 kg flywheel. Net crank torque and angular velocity were sampled at 500 Hz; mechanical power output and cadence were recorded at 10 Hz. Participants used their own cycling shoes and pedals, and individual bike geometry was replicated on the ergometer.

Pulmonary gas exchange (V̇O₂, V̇CO₂) was measured continuously using a breath-by-breath metabolic system (MetaMax 3B, Cortex, Leipzig, Germany), calibrated before each test with certified gas mixtures and a 3 L syringe.

Capillary blood lactate concentration was determined from 20 µL samples obtained from the hyperemic earlobe at rest and 1, 3, 5, 7, 10, 15, 20, 25, and 30 min post-exercise using an enzymatic-amperometric analyzer (Biosen C-Line, EKF Diagnostics, Germany). Muscle oxygenation (SmO₂) of the vastus lateralis was recorded continuously using near-infrared spectroscopy to derive the time constant of phosphocreatine dephosphorylation (τ_PCr_) from post-exercise SmO₂ recovery kinetics (Dunst et al. 2023a). Maximal blood lactate concentration (BLCmax) was determined by fitting a Bateman function to the post-exercise lactate time course to account for the peak occurring between discrete sampling points.

## Gold-standard vLamax assessment (GS)

For the 12 s sprint, the maximal lactate accumulation rate was calculated according to the classical formulation proposed by Mader:

$$\:vLama\mathrm{x},\mathrm{G}\mathrm{S}=\frac{{\Delta\:}BLC}{T-{t}_{\mathrm{alac}}},$$

where ΔBLC denotes the net increase in capillary blood lactate concentration, T the effective sprint duration (~ 12 s), and talac the initial alactic phase as the time delay of lactate accumulation onset (Mader 1994).

Fatigue-free force–velocity (F–v) profiles were derived from the first 3–4 pedal strokes (≤ 3 s) of each sprint, supplemented by a low-resistance sprint at pedalling rates above 160 rpm (Dunst et al. 2022). The time of first systematic deviation from the individual F–v profile (tFF) was used to operationally define the alactic phase duration (talac).

### Indirect determination of vLamax: energy-share framework

**Fig. 1:**
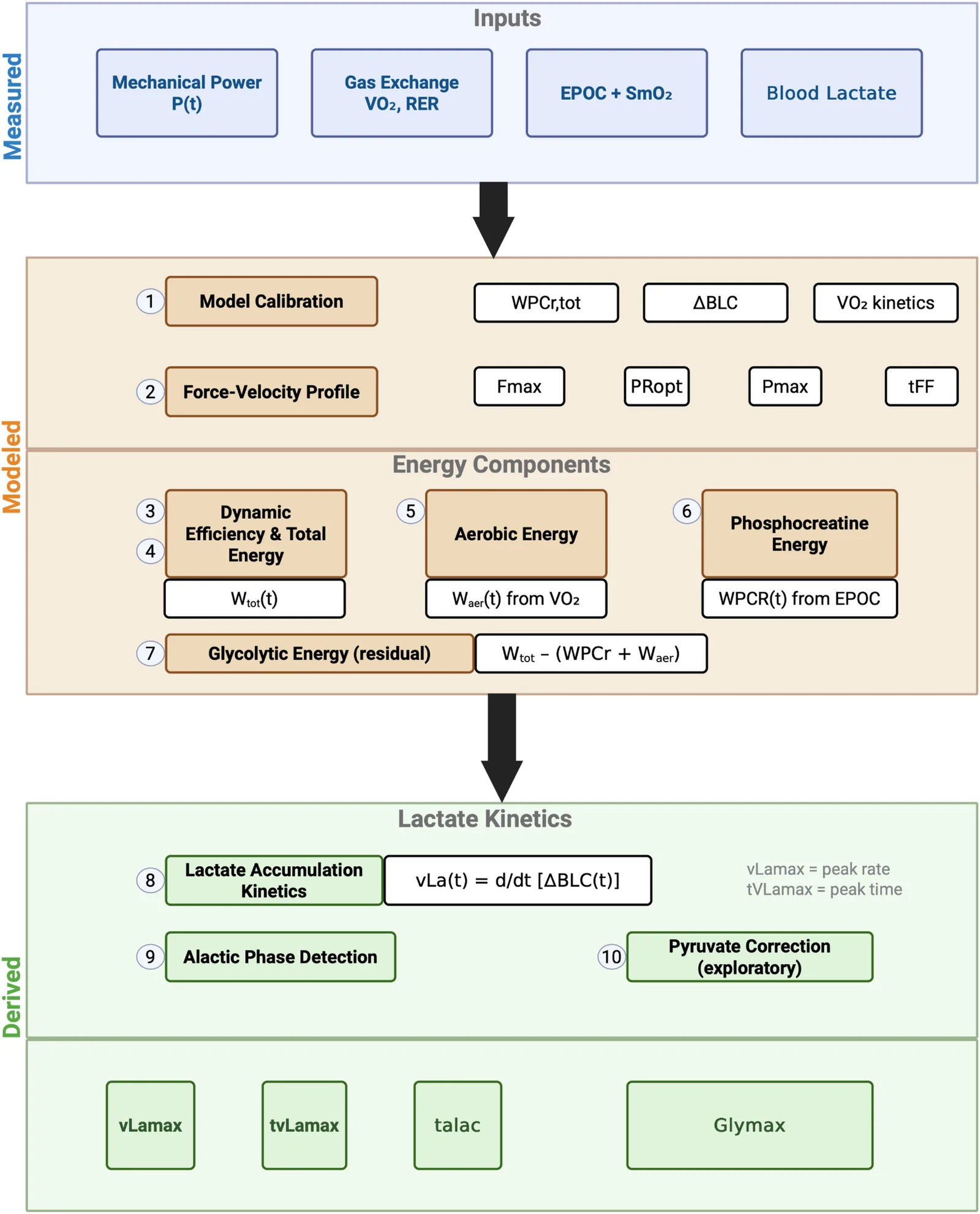
Schematic overview of the ten-step computational workflow for indirect, time-resolved estimation of vLamax. The figure uses three colour categories. Blue boxes indicate measured inputs (mechanical power, gas exchange, EPOC with muscle oxygenation, blood lactate). The warm-coloured modelled section comprises the three independent calibration anchors and the force–velocity profile together with the modelled energy components (dynamic efficiency and total metabolic energy, aerobic energy, phosphocreatine energy) and the glycolytic residual. Green indicates derived results, including the primary outputs vLamax, tvLamax, talac, and Glymax

Time-resolved lactate accumulation kinetics were reconstructed using a three-component energy-share approach (Di Prampero 2005). At any time t during the sprint, total metabolic energy turnover is partitioned as:

1 $$\:{W}_{TOT}\left(t\right)={W}_{PCr}\left(t\right)+{W}_{BLC}\left(t\right)+{W}_{AER}\left(t\right)$$

where WPCr(t) denotes phosphocreatine-derived alactic energy, WBLC(t) glycolytic energy associated with lactate formation, and WAER(t) aerobic oxidative energy. Since WBLC(t) cannot be measured directly, it was obtained as the residual after independent reconstruction of the remaining components. The full computational workflow is illustrated in Fig. 1. The reconstruction was performed entirely on the 60 s maximal sprint, which also provided the three independent calibration anchors: (i) total phosphocreatine capacity from EPOC, (ii) net blood lactate accumulation (ΔBLC), and (iii) breath-by-breath pulmonary gas-exchange kinetics. The 12 s sprint was not reconstructed; it gave the gold-standard vLamax (Mader formula) and an independently measured ΔBLC(12), used to validate the model’s predicted cumulative lactate at 12 s.

*Model calibration.* To ensure physiological identifiability, the model was constrained by three independent experimental anchors derived from the 60 s sprint: (i) total phosphocreatine capacity (WPCr, TOT), obtained from the fast component of a bi-exponential fit to excess post-exercise oxygen consumption (30 min passive recovery) using a caloric equivalent of 20.9 J/ml (Knuttgen 1970; Rossiter et al. 2002; Beneke et al. 2004; Scott 2005); (ii) net blood lactate accumulation (ΔBLC), measured from capillary blood samples; and (iii) continuous breath-by-breath pulmonary gas exchange kinetics.

Dynamic gross efficiency and total metabolic energy. During maximal sprints, gross efficiency declines with fatigue and cannot be assumed constant. Efficiency η(t) was modeled as a mono-exponential function constrained by two anchor points: (i) efficiency at sprint onset, derived from the initial mechanical power output and early V̇O₂ kinetics, and (ii) efficiency at the end of the 60 s sprint, derived from concurrent pulmonary gas exchange. The temporal shape of η(t) was aligned with the experimentally observed decay of maximal power output (Ettema and Loras 2009; Dunst et al. 2023d). Total metabolic energy turnover was modeled as a saturating exponential:

2 $${W_{TOT}}\left( t \right){\rm{ }} = {\rm{ }}{A_W}(1 - {e^{\frac{{ - t}}{{{\tau _W}}}}})$$

where A_W_ represents the asymptotic maximal energy supply and τ_W_ the corresponding time constant (in the denominator of the exponent). AW and τ_W_ were obtained by fitting this mono-exponential to anchor points derived from the 60 s sprint, following the dynamic-efficiency procedure of Dunst et al. (2023d), where the derivation of η(t) is given in full. Instantaneous metabolic power was obtained as:

3 $$MP\left( t \right) = \frac{{P\left( t \right)}}{{\eta \left( t \right)}} = \frac{{d{W_{TOT}}}}{{dt}}$$

Aerobic energy. WAER(t) was quantified directly from measured gas exchange without kinetic modeling. Breath-by-breath V̇O₂(t) and RER(t) were temporally aligned, interpolated to a uniform time grid, and integrated:

4 $${W_{AER}}\left( t \right) = \mathop \smallint \limits_0^{60} VO_{2}\left( t \right)\;\cdot\;CE\left( {RER\left( t \right)} \right)\;dt$$

where the caloric equivalent CE(RER) = 5.157 ∙ RER + 15.974 (J/ml) for RER < 1.0 and 21.131 J/ml for RER ≥ 1.0 (Di Prampero 2005).

*Phosphocreatine energy.* WPCr(t) was reconstructed as a saturating mono-exponential:

5 $${W_{PCr}}\;\left( t \right) = {W_{PCrTOT}}\; \cdot \left( {1 - {e^{\frac{{ - t}}{{{\tau _{PCr}}}}}}} \right)$$

where W_PCr, TOT_ was derived from EPOC (see calibration above) and τ_PCr_ was set equal to the time constant (τSmO₂) of a mono-exponential fit to the post-exercise muscle-oxygenation (SmO₂) recovery measured via near-infrared spectroscopy (Hamaoka et al. 2007; Ryan et al. 2012; Dunst et al. 2023a).

*Glycolytic energy and lactate kinetics.* Glycolytic energy was isolated as:

6 $$W_{BLC} (t)=W_{TOT} (t)-W_{PCr} (t)-W_{AER} (t)$$

Cumulative blood lactate accumulation was derived from glycolytic energy:

7 $${\rm{\Delta }}BLC\left( t \right) = {W_{BLC}}\left( t \right) \cdot LA{O_2}^{ - 1} \cdot B{M^{ - 1}} \cdot CE{(RER)^{ - 1}}$$

where LAO₂ = 3.0 ml O₂·kg⁻¹·(mmol/L)⁻¹ is the oxygen–lactate equivalent assuming a lactate distribution volume of 50% of body mass (Di Prampero 2005), CE = 21.131 J/ml the caloric equivalent at RER ≥ 1.0, and BM the participant’s body mass. The instantaneous lactate accumulation rate was obtained by temporal differentiation:

8 $$vLa\left( t \right) = \frac{d}{{dt}}\left[ {\Delta BLC\left( t \right)} \right]$$

The maximal lactate accumulation rate was defined as vLamax = max[vLa(t)], with the corresponding time point denoted tvLamax.

*Alactic phase detection.* The onset of glycolytic energy contribution was identified as the first time point at which W_BLC_(t) remained continuously positive for at least 0.5 s, marking the end of the initial lactate-free alactic phase (talac).

Oxidative pyruvate correction (exploratory). During maximal exercise, a fraction of glycolytically produced pyruvate is concurrently oxidized in the mitochondria rather than reduced to lactate. To approximate total glycolytic flux (Glymax) independent of pyruvate fate, the measured aerobic energy contribution was used to estimate the oxidative pyruvate disposal rate. Based on the stoichiometric relationship between oxygen consumption and pyruvate oxidation (Mader 2003), the concurrent oxidative disposal was estimated from the fraction of muscular V̇O₂ directed toward pyruvate oxidation, yielding Gly(t) = vLa(t) + vLaox(t) and Glymax = max[Gly(t)]. This approach represents a first-order approximation, as the actual rate of pyruvate oxidation depends on intracellular lactate concentration and pyruvate dehydrogenase activity, which vary across individuals and exercise intensities (Mader 2003; Parolin et al. 1999).

An openly accessible simulation tool implementing the full model is available at https://benemeixner.shinyapps.io/vlamax-simulation/to facilitate reproducibility and applied use.

### Statistical analysis

Data processing was conducted using Microsoft Excel 2016 and Python, while statistical analyses were performed in JASP (Version 0.18.2, University of Amsterdam). Data distributions were assessed prior to hypothesis testing. Normality was evaluated using the Shapiro-Wilk test, and homogeneity of variances using Levene’s test. Descriptive statistics are reported as mean ± standard deviation unless otherwise noted.

Bivariate relationships between physiological and performance variables were analyzed using Pearson correlation coefficients for normally distributed data and Spearman rank correlations for non-normal distributions. Paired t-tests were employed to compare vLamax values between methods, with effect sizes reported as Cohen’s d (paired: mean difference/SD of differences). Agreement between predicted and measured values was assessed using Bland-Altman analysis (mean bias, 95% limits of agreement, proportional bias) and standard error of estimate (SEE).

Stepwise multiple linear regression was used to identify key predictors of selected physiological and performance outcomes. Model assumptions, including linearity, normality of residuals, homoscedasticity, and absence of multicollinearity, were systematically verified following established guidelines (Osborne and Waters 2002). Regression outputs include adjusted coefficients of determination (adjusted R²), standardized β coefficients, F-statistics, and associated p-values. To assess the predictive utility of the indirect method for correcting gold-standard estimates, a linear calibration model was derived from bivariate regression of vLamax, IA on vLamax, GS. Model stability was evaluated using leave-one-out cross-validation (LOO-CV). Statistical significance was set at α < 0.05.

Sensitivity of vLamax, IA to fixed model parameters and kinetic assumptions was assessed by systematic variation of each parameter within physiologically plausible bounds while monitoring effects on primary outcomes and ΔBLC prediction accuracy. For LAO₂, the reference value (3.0 ml O₂·kg^−1^·(mmol/L)^−1^) was varied by ± 10%, and the effect on both vLamax, IA and predicted ΔBLC(12) was recorded; because vLamax, IA scales linearly with 1/LAO₂, ΔBLC prediction accuracy serves as an empirical constraint on admissible LAO₂ values. The assumed fraction of oxidative pyruvate disposal was varied by ± 25% to assess its influence on the exploratory Glymax estimate. To assess sensitivity to the assumed kinetic form of phosphocreatine depletion – the model parameter with the greatest potential influence on both vLamax, IA and tvLamax – τ_PCr_ was perturbed by ± 20% and ± 30% around its individually calibrated value, and the mono-exponential WPCr(t) was replaced with bi-exponential alternatives spanning a range of fast-to-slow component ratios (80:20 and 20:80). For each perturbation, the magnitude and direction of the IA–GS difference were recorded as the primary outcome criterion, with preservation of the direction of bias considered the minimum requirement for qualitative robustness. To test whether body-composition heterogeneity distorts the reconstructed peak, vLamax, IA was additionally recomputed with the lactate distribution space scaled to fat-free mass (FFM) instead of total body mass, with the oxygen–lactate equivalent re-anchored to preserve the empirically constrained cohort ΔBLC(12); because tvLamax and talac are determined by the temporal shape of WBLC(t) and are analytically invariant to any constant scaling factor, this analysis concerns the peak height only.

## Results

### Inter-individual variability and glycolytic kinetics

The 60 s all-out sprint elicited maximal post-exercise blood lactate concentrations of 18.6 ± 2.8 mmol/L, confirming exhaustive glycolytic engagement across all participants. Within this uniformly high metabolic stress, the indirect assessment revealed pronounced inter-individual differences in glycolytic performance: vLamax, IA was 0.91 ± 0.18 mmol/L/s (range: 0.57–1.23). As an exploratory extension, correction for concurrent oxidative pyruvate utilization yielded estimated maximal glycolytic flux (Glymax) of 1.13 ± 0.16 mmol/L/s, approximately 24% higher than vLamax (*p* < 0.001, Cohen’s d = 2.52), indicating a substantial fraction of glycolytic flux not captured by blood lactate appearance alone.

The ratio of maximal glycolytic flux to maximal lactate accumulation rate (Glymax/vLamax) showed pronounced inter-individual variability (range: 1.09–1.76; mean: 1.26 ± 0.16). A trend toward higher ratios was observed in athletes with greater aerobic capacity (*r* = 0.44, *p* = 0.074), suggesting that a larger fraction of glycolytic pyruvate is concurrently oxidized in athletes with higher V̇O₂peak.

**Fig. 2:**
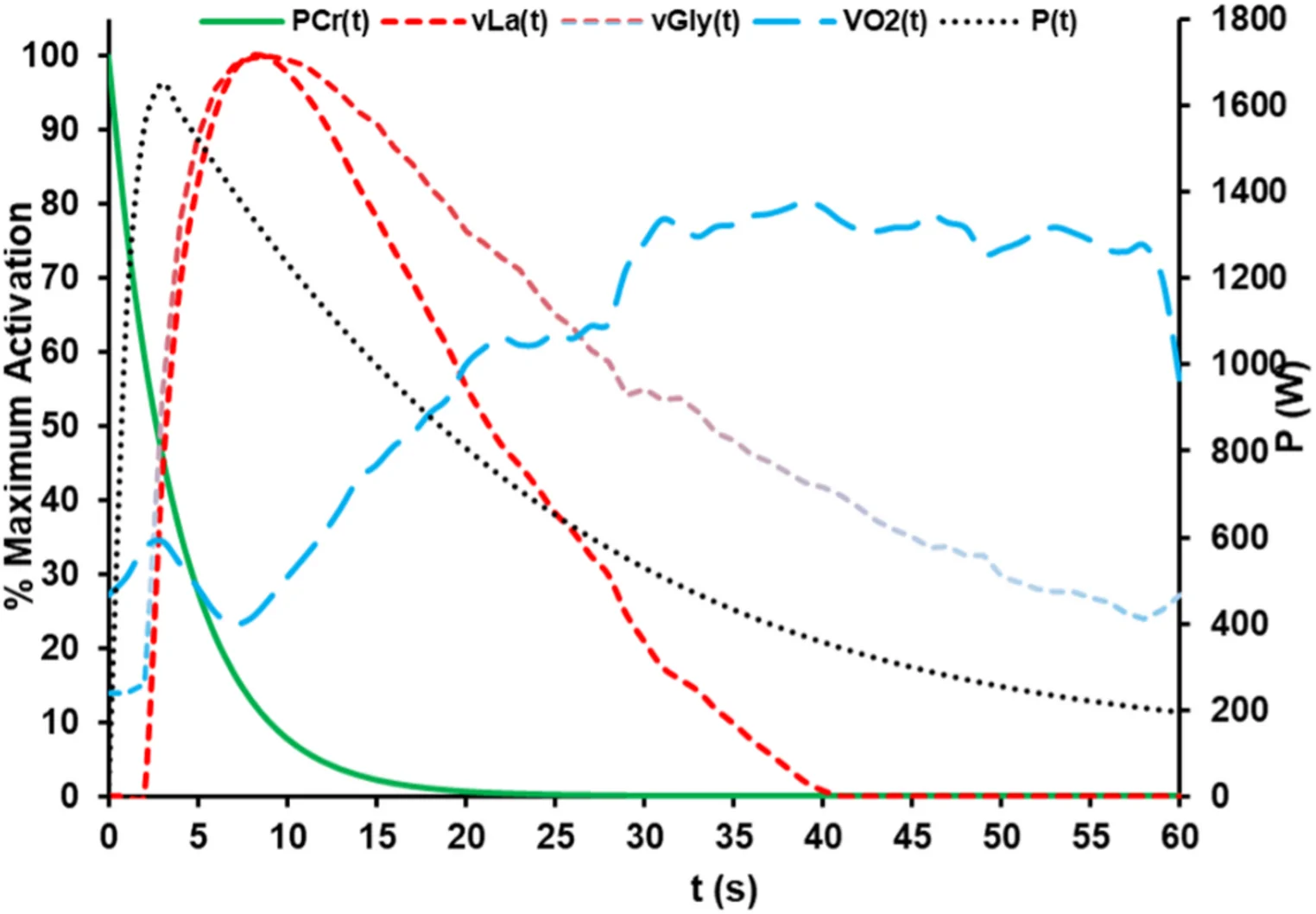
Time course of metabolic system activation during an individual maximal 60 s sprint (selected to illustrate typical temporal dynamics; individual peak timing may differ from the group mean), expressed as percentage of individual maximum. Phosphocreatine (PCr, green) depletes rapidly from sprint onset; glycolytic rate (vLa, red) peaks at approximately 8 s before declining due to pH-mediated self-inhibition; total glycolytic flux including oxidative pyruvate disposal (vGly, rendered as a gradient from red toward blue to depict the progressive shift from glycolytic to oxidative pyruvate disposal) peaks slightly later; aerobic energy supply (V̇O₂, blue) rises progressively; mechanical power output (P, black dotted) declines monotonically

**Fig. 3:**
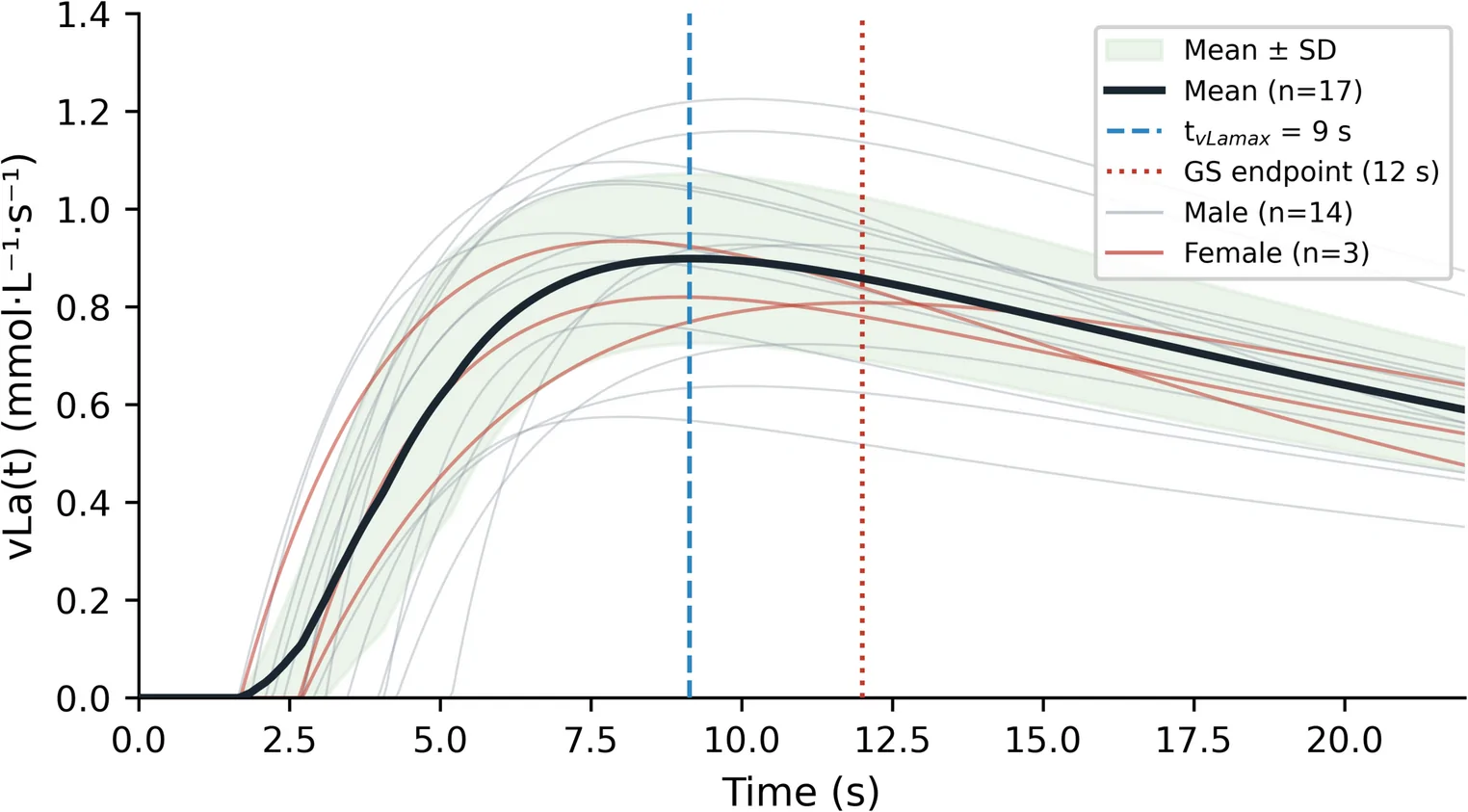
Individual time courses of instantaneous lactate accumulation rate vLa(t) during the first 22 s of the 60 s sprint for all 17 participants. Curves represent bi-exponential Bateman functions fitted to individual model parameters (vLamax, tvLamax, tFF, ΔBLC₁₂). Group mean (black line) and mean ± SD (green shading). Male participants (*n* = 14) in gray, female participants (*n* = 3) in red. Dashed blue line: mean time to peak glycolytic rate (tvLamax = 9 s); dotted red line: conventional 12 s gold-standard endpoint

Temporal characteristics further highlighted marked inter-individual variability. Time to peak lactate accumulation rate (tvLamax) spanned 7–12 s, and the modeled alactic phase duration (talac) ranged from 1 to 5 s. Glycolytic activation was sharply time-confined: at least 90% of vLamax was sustained for ~ 4 s around the peak, with a central ~ 2 s interval exceeding 95% activation for all participants. Representative time courses of power output, V̇O₂ kinetics, PCr dephosphorylation, glycolytic energy contribution, and cumulative blood lactate accumulation during a 60 s sprint are shown in Figs. 2 and 3.

### Validation against the gold-standard protocol

**Fig. 4:**
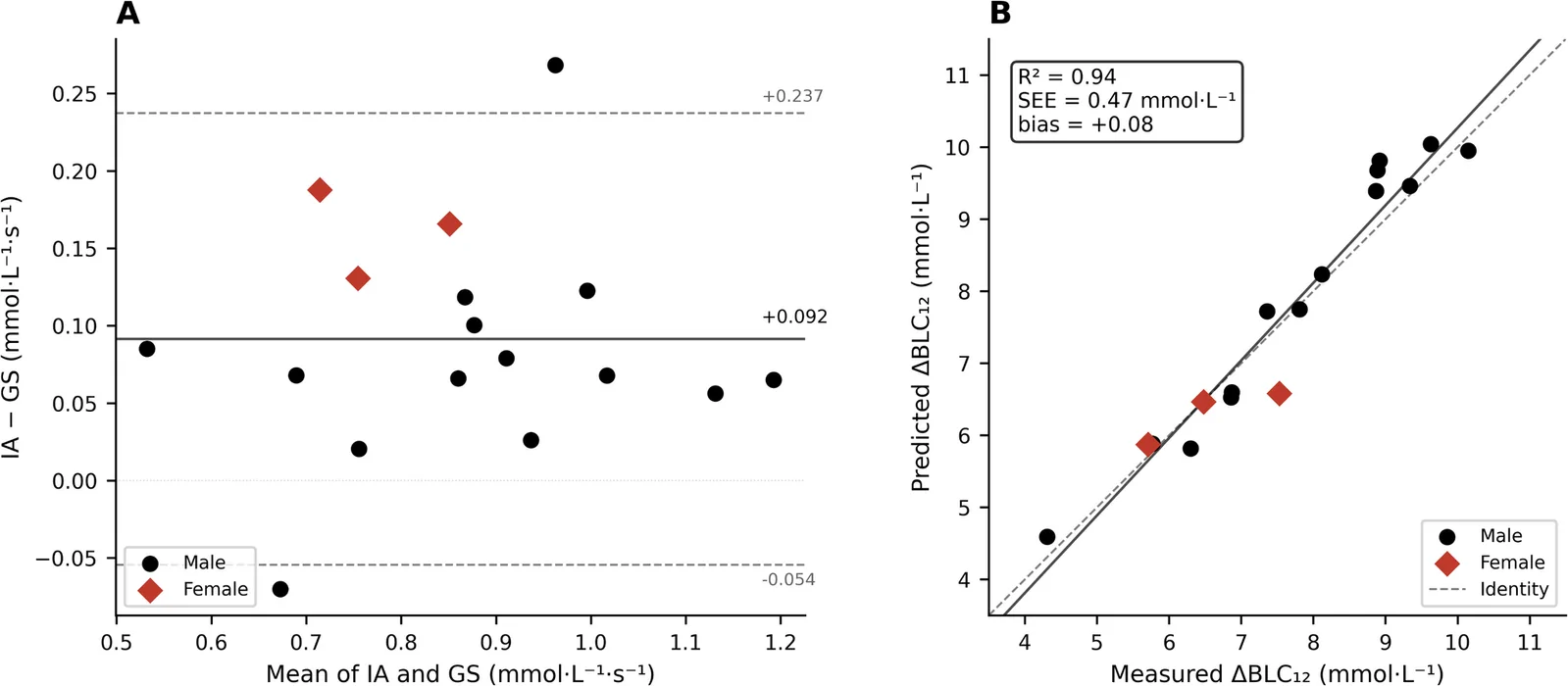
Method comparison between indirect assessment (IA) and gold-standard (GS). **A** Bland–Altman plot for vLamax: mean bias + 0.092 mmol·L^−1^·s^−1^; 95% limits of agreement − 0.054 to + 0.237 mmol·L^−1^·s^−1^. No proportional bias (*r* = 0.10, *p* = 0.706). **B** Predicted versus measured cumulative blood lactate at 12 s (ΔBLC₁₂). R^2^ = 0.94, SEE = 0.47 mmol·L ^−1^, mean bias + 0.08 mmol/L. Dashed line indicates identity; solid line indicates linear regression. Black circles: male (*n* = 14); red diamonds: female (*n* = 3)

Despite higher absolute vLamax values, the indirect method reproduced the gold-standard outcome for cumulative lactate accumulation. Predicted ΔBLC(12) (7.67 ± 1.77 mmol/L) closely matched measured values (7.58 ± 1.60 mmol/L; *p* = 0.464, Cohen’s d = 0.18), with a standard error of estimate (SEE) of 0.47 mmol/L (Fig. 4). In contrast, vLamax, IA was systematically higher than vLamax, GS (0.91 ± 0.18 vs. 0.82 ± 0.17 mmol/L/s; *p* < 0.001; Cohen’s d = 1.23), with an SEE of 0.13 mmol/L/s (Fig. 4).

Bland-Altman analysis confirmed the absence of systematic bias for ΔBLC(12) (mean difference: 0.08 mmol/L; 95% limits of agreement [LoA]: −0.82 to 0.98 mmol/L), with no significant proportional bias (*r* = 0.39, *p* = 0.123). For vLamax, a systematic positive bias of 0.09 mmol/L/s was observed (95% LoA: −0.05 to 0.24 mmol/L/s). No proportional bias was detected (*r* = 0.10, *p* = 0.705), indicating that the magnitude of disagreement was independent of glycolytic capacity (Fig. 4).

To enable retrospective correction of gold-standard estimates, a linear calibration model was derived: vLamax, IA = 0.95 × vLamax, GS + 0.13 (R² = 0.83, SEE = 0.08 mmol/L/s). Leave-one-out cross-validation confirmed model stability (R² = 0.79, RMSE = 0.08 mmol/L/s).

Sensitivity analysis examined the influence of fixed model parameters and kinetic assumptions on vLamax, IA. Absolute values scaled linearly with 1/LAO₂, such that a ± 10% change in LAO_2_ produced a corresponding ± 10% change in vLamax, IA. However, LAO₂ was empirically constrained by the close agreement between predicted and measured ΔBLC(12): deviations from the reference value (3.0 ml O_2_·kg⁻¹·(mmol/L)^−1^) produced significant discrepancies in ΔBLC prediction (*p* < 0.001 for ± 10% LAO_2_). The assumed fraction of oxidative pyruvate disposal affected only the exploratory Glymax estimate (Glymax/vLamax ratio range: 1.18–1.29 for ± 25% variation) and did not influence vLamax or tvLamax. To assess sensitivity to the assumed kinetic form of phosphocreatine depletion, vLamax and tvLamax were recomputed after varying τ_PCr_ by ± 20–30% and after replacing the mono-exponential WPCr(t) with bi-exponential alternatives (fast/slow component ratios of 80/20 and 20/80). Perturbation of τ_PCr_ by ± 20% shifted vLamax by + 6.4%/−4.4%, while ± 30% produced + 10.6%/−3.8% shifts. Bi-exponential forms increased vLamax by 14–16% and shifted tvLamax earlier by 2–5 s. The qualitative finding that IA exceeds GS was preserved under all perturbations (IA–GS difference: +3.0% to + 24.8%), confirming that the direction of the systematic bias is robust to kinetic model choice. However, the absolute magnitude of vLamax and particularly tvLamax are moderately dependent on the assumed WPCr kinetics. Re-scaling the lactate distribution space to fat-free mass rather than total body mass shifted individual vLamax, IA by less than 5% for 16 of 17 participants (mean 0%); only the single participant with markedly higher body fat (23.5%) shifted by 12%. The cohort mean and SD were unchanged (SD 0.177 → 0.178 mmol/L/s), the between-athlete rank order was preserved (Spearman ρ = 0.97), body fat was uncorrelated with vLamax (*r* = − 0.05), and peak timing was unaffected (invariant by construction). Fat-free mass is therefore the more appropriate denominator in cohorts with higher or more variable body fat, whereas in the present low-fat sample the choice of denominator does not affect the results. Re-running the primary comparisons under FFM scaling confirmed this: the vLamax, IA–vLamax, GS difference remained significant and of the same magnitude (0.91 vs. 0.82 mmol/L/s, + 11%, *p* < 0.001, d = 1.0), and the ΔBLC(12) validation was preserved (bias + 0.07 mmol/L, SEE 0.46 mmol/L, *p* = 0.50).

### Timing of maximal lactate accumulation and validation of alactic phase detection

Peak vLamax occurred significantly earlier than the fixed 12 s endpoint of the gold-standard test. On average, tvLamax was 9.12 ± 1.41 s (range: 7–12 s), substantially preceding the assumed 12 s sampling window (*p* < 0.001, Cohen’s d = − 2.05). This dispersion (± 1.4 s) reflects between-athlete variability at a fixed model form, not the precision of the group mean: in the sensitivity analysis, bi-exponential phosphocreatine kinetics shifted the mean tvLamax by up to ~ 5 s. The exact peak timing should therefore be interpreted as approximate, although its occurrence before the 12 s endpoint was robust across all kinetic forms tested. As an independent validation of the modeled alactic phase, the mechanically derived time of first deviation from the fatigue-free force–velocity profile (tFF = 2.92 ± 0.99 s) was compared with the energy-balance–derived glycolytic onset (talac = 2.55 ± 0.94 s). The two estimates showed close numerical agreement and were not significantly different (*p* = 0.10) though the sample size limits the power to detect small systematic offsets.

### Physiological correlates of vLamax, tvLamax, and tFF

**Fig. 5:**
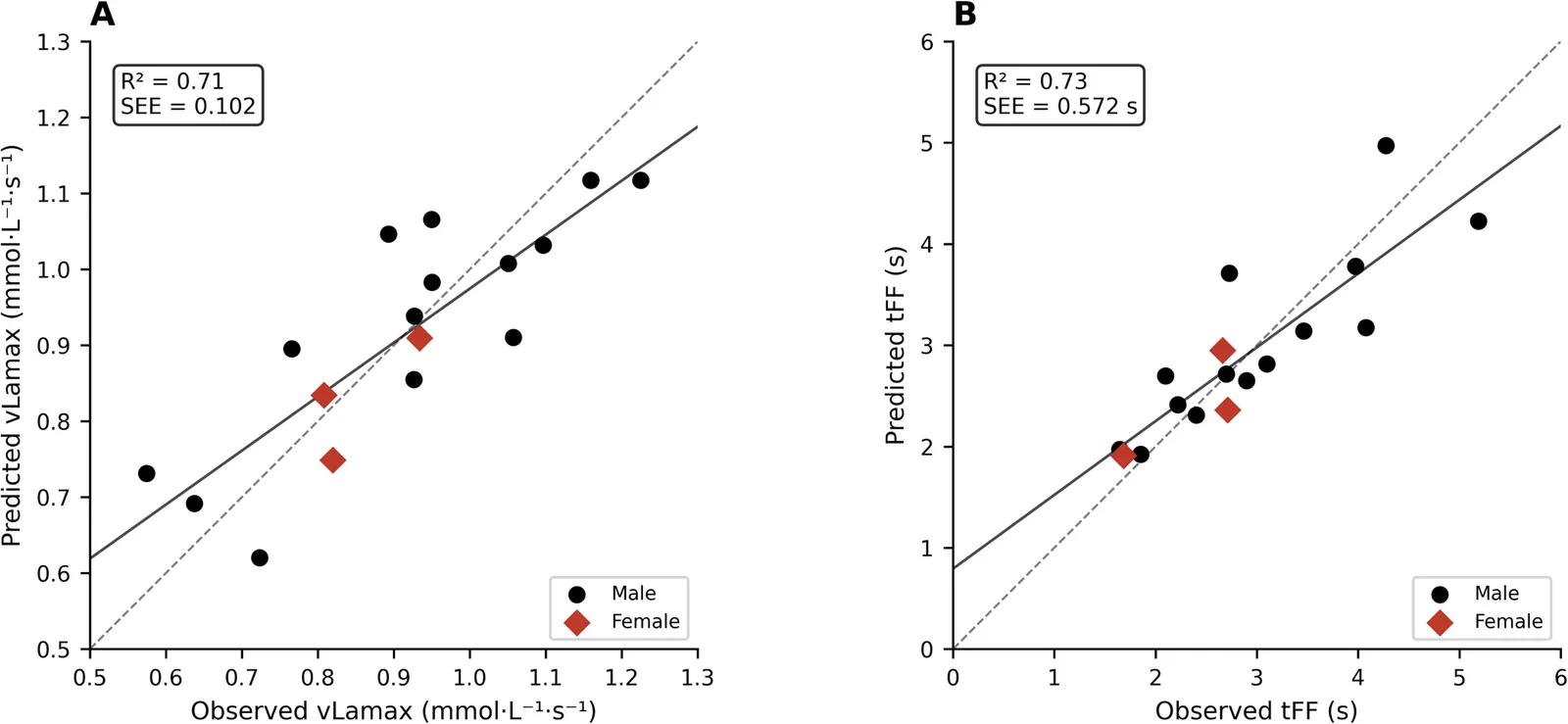
Predicted versus observed values for key model outcomes. Black circles: male (*n* = 14); red diamonds: female (*n* = 3). **A** vLamax predicted from maximal isometric force (Fmax) and relative aerobic capacity (V̇O₂peak, rel); R² = 0.71, SEE = 0.102 mmol·L⁻¹·s⁻¹. **B** Alactic phase duration (tFF) predicted from V̇O₂peak, rel, the slope of the linear force–velocity relationship, and PCr; R² = 0.73, SEE = 0.57 s. Each point represents one participant; vertical distance from the identity line indicates the model’s prediction error. Dashed lines indicate identity; solid lines indicate linear regression

BLCmax was the strongest statistical correlate of vLamax, IA (BLCmax dominant, β = +0.740, *p* < 0.001; R² = 0.879, adjusted R² = 0.852). This association is partly built in, however, because vLamax, IA is reconstructed in part from the blood-lactate data and peak lactate is itself a downstream consequence of a high glycolytic rate. BLCmax is therefore a downstream marker rather than an independent physiological determinant, and we do not interpret it mechanistically. The analysis of independent determinants therefore excluded BLCmax, in which maximal isometric force (Fmax) and relative aerobic capacity (V̇O₂peak, rel) formed the best-fitting two-predictor model (R² = 0.711, adjusted R² = 0.669; RMSE = 0.102 mmol/L/s; F(2,14) = 17.21, *p* < 0.001). Fmax was positively associated with vLamax (β = +0.779, *p* < 0.001), while V̇O₂peak, rel was negatively associated (β = −0.397, *p* = 0.016). In an exploratory subgroup analysis restricted to sprint specialists (V̇O₂peak, rel ≤ 60 ml/kg/min; *n* = 11), Fmax alone explained 74.4% of vLamax variance (R² = 0.744, adjusted R² = 0.715; SEE = 0.079 mmol/L/s; F(1,9) = 26.11, *p* < 0.001), while the addition of V̇O₂peak, rel did not improve model fit (*p* = 0.607). The predicted-versus-observed plot for this model is shown in Fig. 5A.

For tvLamax, exploratory stepwise regression retained optimal pedalling rate (PRopt) and phosphocreatine capacity (PCr) as significant predictors (R² = 0.493, adjusted R² = 0.420; RMSE = 1.07 s; F(2,14) = 6.79, *p* = 0.009). PRopt was negatively associated with tvLamax (β = −0.792, *p* = 0.003) and PCr was positively associated (β = +0.549, *p* = 0.026). For tFF, an exploratory three-predictor model (V̇O₂peak, rel, the slope of the linear force–velocity relationship, and PCr) explained 72.9% of variance (adjusted R² = 0.666; RMSE = 0.57 s; F(3,13) = 11.65, *p* < 0.001), with V̇O₂peak, rel as the dominant predictor (β = +0.992, *p* < 0.001). The predicted-versus-observed plot for the tFF model is shown in Fig. 5B. In these predicted-versus-observed plots each point is one participant, the dashed line is the line of identity, and the solid line the regression fit, so vertical distance from identity indicates the model’s prediction error.

## Discussion

The present findings show that a time-resolved energy-share approach enables physiologically consistent indirect estimation of maximal lactate accumulation rate from a single maximal sprint. The close agreement between predicted and measured 12 s blood lactate accumulation confirms the physiological consistency of the three-component energy partitioning and supports the validity of the reconstruction framework (Di Prampero 2005; Knuttgen 1970; Beneke et al. 2002). This validation concerns cumulative lactate accumulation. The resolved peak rate and its timing are model-derived and examined below.

A key methodological contribution is to address the two dominant limitations of conventional vLamax testing: rather than correcting for an assumed alactic delay or selecting an arbitrary sprint duration, the time-resolved approach derives both the glycolytic onset and the peak rate directly from the reconstructed energy trajectory, making their estimation an output of the model rather than a prerequisite input (Dunst and Hesse 2023e; Langley et al. 2025). The model provides independent evidence that an initial lactate-free phase exists under maximal sprint conditions: glycolytic contribution remained negligible during the first ~ 1–4 s, consistent with predominant phosphocreatine coverage at sprint onset. The close correspondence between modeled talac and the mechanically derived fatigue-free time span supports construct validity of this early alactic phase. Moreover, the exploratory regression model for tFF (see Results) suggests a potential estimation pathway when fatigue-free profiling is not feasible.

Once talac is addressed with physiologically defensible methods, the dominant limitation of current gold-standard protocols becomes apparent: vLamax, GS represents a mean rate averaged over the full glycolytic window rather than an instantaneous peak. Any protocol that extends beyond the point of maximal glycolytic flux will underestimate vLamax by averaging the peak with its post-peak decline. In the present cohort, this averaging window extended approximately 3 s beyond the mean tvLamax of 9 s. This duration was sufficient to capture the steepest portion of the post-peak decline and to produce the observed 10% underestimation. The narrow window of near-maximal glycolytic activation (~ 2–4 s around the peak) further illustrates why even modest extensions of sprint duration carry disproportionate consequences for the accuracy of fixed-duration estimates. A key interpretive question is whether the observed IA–GS difference of ~ 10% reflects genuine underestimation by the gold-standard protocol or systematic overestimation introduced by model assumptions. Two converging lines of evidence support the former interpretation. First, the ΔBLC(12) validation constrains the integral of vLa(t) over the 12 s sprint: because the model correctly reproduces cumulative lactate accumulation, it cannot systematically inflate the total glycolytic energy budget. An overestimating model would necessarily predict higher ΔBLC(12) than measured, which was not observed (mean bias 0.08 mmol/L, *p* = 0.464). Second, sensitivity analysis showed that the direction of the IA–GS difference was preserved across all perturbations of model assumptions, including alternative PCr kinetic forms that shifted vLamax by up to 16%. The magnitude of the difference varied, but it did not reverse. Neither line is conclusive in isolation, but their convergence supports the interpretation that the IA–GS difference reflects a real and directional limitation of fixed-duration protocols rather than an artifact of the indirect model.

Langley et al. (2024) reported that a 10 s sprint duration elicited significantly higher vLamax than 15–30 s durations, and Porter and Langley (2025) reached a similar conclusion using muscle-oxygen-saturation kinetics instead of lactate sampling. The review by Quittmann (2025) summarized the current evidence on test methodology, and Wackerhage et al. (2025) recommended test durations of 10–15 s based on the available literature. The present energy-share approach complements this body of evidence by providing individual, time-resolved lactate kinetics rather than comparisons across fixed sprint durations.

Decomposition shows that window-averaging is the directional component of the IA–GS difference: after appropriate talac correction (which accounts for approximately 22% of the uncorrected GS value; Dunst and Hesse 2023e), a residual underestimation of approximately 10% persisted and is attributable to temporal averaging across the post-peak decline of glycolytic activity. Uncertainty in the alactic correction itself adds non-directional variability but is not a consistent bias. Protocol refinements that address only talac therefore cannot resolve this systematic component.

The observed temporal profile is mechanistically grounded in the Mader framework (Mader 2003), in which glycolytic flux is shaped by the opposing dynamics of ADP-mediated phosphofructokinase activation and plausibly hydrogen-ion-mediated self-inhibition, among other feedback effects. At sprint onset, rapid phosphocreatine depletion elevates cytosolic ADP, activating glycolysis. As lactate accumulates, the concomitant fall in intramuscular pH progressively inhibits phosphofructokinase, reducing glycolytic flux despite continued ADP elevation. The peak of vLa(t) thus represents the transient instant at which activation maximally exceeds inhibition, a point determined by the individual balance between phosphocreatine buffering capacity, glycolytic enzyme capacity, and intracellular buffer capacity (Erecińska et al. 1995; Gastin 2001; Mader 2003).

Exploratory regression analyses suggested distinct physiological determinants for the magnitude and timing of peak glycolytic activity. The distinction between causal determinants and downstream consequences must be considered when interpreting these associations. BLCmax, while statistically the strongest predictor, is itself a consequence of high glycolytic rate rather than an independent determinant: athletes with higher vLamax necessarily accumulate more lactate, rendering this association circular. Its strong predictive role therefore confirms internal consistency of the indirect assessment rather than revealing a mechanistic driver. When this non-causal predictor was excluded, the physiologically interpretable model retained maximal isometric force (Fmax) and relative aerobic capacity (V̇O₂peak, rel). In the present sprint cycling cohort, the positive association between Fmax and vLamax may reflect covariation of neuromuscular and glycolytic capacity under sprint-specific training, in which force development and glycolytic enzyme expression in type II fibres tend to increase together. We offer this as an interpretation rather than a demonstrated mechanism. This coupling is not obligatory, as high force production can occur without elevated glycolytic capacity in athletes from non-glycolytic training backgrounds. Nitzsche et al. (2018) reported no relationship between maximal power and vLamax in either isokinetic leg press or cycle sprint tests in a heterogeneous athletic cohort, confirming that neuromuscular force and glycolytic capacity are independent traits in the absence of sprint-specific training. Within the homogeneous training context of elite track sprinters, however, concurrent development of both capacities produces a robust statistical association. The subgroup analysis supports this interpretation: when restricted to sprint specialists (V̇O₂peak, rel ≤ 60 ml/kg/min, *n* = 11), Fmax alone explained the majority of vLamax variance, whereas V̇O₂peak, rel contributed no additional predictive value. This suggests that within a training-homogeneous sprint cohort, neuromuscular force capacity serves as a sufficient proxy for glycolytic capacity, while the negative contribution of V̇O₂peak, rel in the full sample primarily reflects the inclusion of athletes with higher aerobic profiles and correspondingly greater concurrent oxidative pyruvate disposal.

For peak timing, athletes with higher optimal pedalling rate reached peak glycolytic activation earlier, consistent with faster neuromuscular activation, whereas greater phosphocreatine capacity delayed the glycolytic peak (Mader 2003; Dunst et al. 2023b), consistent with suggestive results from reanalysis of the data in Meixner et al. (2025). The moderate overall predictive accuracy indicates that tvLamax is partly governed by factors not captured by standard performance diagnostics, reinforcing the value of individualized time-resolved assessment.

As an exploratory extension, the framework allows correction of blood lactate accumulation for concurrent oxidative pyruvate utilization, providing a first-order approximation of total maximal glycolytic flux (Glymax) independent of lactate appearance alone (Mader 2003; Yang et al. 2023). The approximately 24% higher Glymax compared with vLamax is consistent with metabolic modeling indicating that approximately 70–80% of glycolytically generated pyruvate accumulates as lactate during supramaximal exercise (Mader 1984, 2003). The Glymax-to-vLamax ratio varied considerably across individuals, with a trend toward higher values in athletes with greater aerobic capacity. This observation suggests that aerobically trained sprint cyclists may oxidize a larger fraction of glycolytic pyruvate, consistent with higher mitochondrial density and pyruvate dehydrogenase activity in oxidative muscle fibers (Parolin et al. 1999). However, because this approximation does not account for individual variation in pyruvate dehydrogenase activity or lactate-dependent oxidation kinetics, Glymax estimates should be interpreted with caution.

A separate question concerns the use of vLamax as input to integrative metabolic models. In Mader’s framework, vLamax denotes the ceiling of glycolytic rate under saturating activation and negligible inhibition. The realized formation peak is lower, and the window-averaged gold-standard value lower still. The effective constants of the Mader model were established against sprint-derived gold-standard inputs and are therefore internally calibrated to that quantity. Whether the reconstructed instantaneous peak is the more appropriate individual input is unclear, because substituting it without re-deriving the model constants could break the calibration. The present framework separates the realized formation peak from the window-averaged blood-appearance rate but cannot, on its own, decide which of the two should enter the model. That decision requires joint estimation of input rate and model parameters against independent performance data.

### Practical applications

The findings support a shift toward kinetics-informed and, where feasible, individualized sprint diagnostics. Accurate determination of vLamax is particularly critical when lactate-based parameters are integrated into physiological or performance models, because small systematic biases propagate into substantial prediction errors. The observed association of vLamax with Fmax and V̇O₂peak, rel provides practitioners with a preliminary framework for interpreting inter-individual differences: athletes combining high neuromuscular force capacity with moderate aerobic power may be expected to exhibit higher lactate accumulation rates within comparable sprint-cycling cohorts. This also implies that training-induced shifts in either component, for instance an increase in aerobic capacity following endurance blocks, may alter vLamax independently of changes in glycolytic enzyme content.

For practitioners using conventional 12 s sprint protocols, the linear correction model derived in the Results provides kinetics-informed estimates without requiring the full energy-share reconstruction. This correction was cross-validated and may serve as a pragmatic interim solution within the population studied (elite track cyclists, *n* = 17). Generalizability to other disciplines or performance levels remains to be established. When fatigue-free force–velocity profiling is available, tFF offers a measurement-based estimate of the alactic phase duration, removing a major source of protocol bias. Where tFF is unavailable, the exploratory tFF regression model (see Results) may approximate the alactic delay from routine diagnostic data. Similarly, because peak blood lactate (BLCmax) is a simple, routinely measured quantity that correlates strongly with vLamax, it may serve as a first-order practical proxy for glycolytic power, although this relationship is partly built into the present reconstruction and its independent validity remains to be confirmed against a vLamax measure not derived from blood lactate.

Shortened or personalized test durations centered around the individual peak glycolytic window may substantially improve diagnostic validity and sensitivity in elite sport (Langley et al. 2024; Wackerhage et al. 2025). The energy-share reconstruction does not require a separate incremental exercise test. All necessary inputs – mechanical power, breath-by-breath gas exchange, EPOC, and capillary blood lactate – are obtained from the maximal sprint effort itself, consolidating the diagnostic burden into a single testing session. Beyond cycling, the presented framework provides a transferable template for time-resolved metabolic diagnostics in continuous sprint-based locomotion tasks.

A caution on software interoperability. Integrative metabolic simulators used in applied practice (e.g. INSCYD and other Mader-derived tools) were calibrated with window-averaged, fixed-duration vLamax inputs, which are the very quantity shown here to underestimate the instantaneous peak by ~ 10%. The corrected instantaneous peak is therefore not a drop-in replacement for those inputs: entering it into existing software without re-deriving the internal model constants could break the calibration and inflate predicted glycolytic flux at submaximal intensities, distorting downstream outputs such as the maximal lactate steady state. Practitioners should continue to use the input for which their software was calibrated (the window-averaged value, or the linear correction above applied consistently within their own calibration) and treat the instantaneous peak as a diagnostic descriptor of glycolytic kinetics rather than as a model input, unless input rate and model constants are jointly re-estimated against independent performance data.

### Limitations and future directions

The methodological complexity of the approach currently restricts application to well-equipped laboratory environments with high-resolution mechanical and metabolic instrumentation. Model accuracy depends on assumptions regarding dynamic efficiency behavior (Dunst et al. 2022), lactate distribution volume (Di Prampero 2005), and clearance kinetics (Mader 2003), which may vary across populations and exercise modalities. Breath-by-breath gas exchange during rapid intensity transitions remains technically challenging and may introduce uncertainty during the initial seconds of exercise (Barstow and Mole 1987). Early breath-by-breath V̇O₂ also informs the efficiency anchor at sprint onset, so uncertainty in the first seconds propagates into the reconstructed total metabolic energy and not only into the comparatively small aerobic term. Direct validation against continuous intravascular lactate measurements is currently lacking and represents an important avenue for future work. Because the aerobic term is obtained by direct breath-by-breath integration rather than kinetic fitting, the only quantity that can be perturbed is measured V̇O₂ itself. At the time of peak glycolytic flux the aerobic contribution is small (3–10% of turnover; Yang et al. 2023), so a ± 20% perturbation of the aerobic and onset-efficiency terms changes vLamax, IA by no more than ~ 2% – an order of magnitude below the WPCr-kinetics uncertainty reported above – and does not reverse the IA–GS difference.

The ΔBLC(12) validation constrains the integral of vLa(t) but not its peak value or timing. As reported in the Results, vLamax and particularly tvLamax are moderately dependent on the assumed temporal shape of WPCr(t), although the direction of the IA–GS difference was preserved throughout. The mono-exponential form used here is a common simplification in exercise physiology, though ³¹P-MRS studies have demonstrated that PCr kinetics may deviate from single-exponential behavior depending on exercise intensity (Rossiter et al. 2002). Alternative parameterizations for maximal sprint conditions cannot be excluded.

A related source of uncertainty concerns the physiological basis of LAO₂ itself. The conventional value of 3.0 ml O₂·kg⁻¹·(mmol/L)⁻¹ embeds a fixed lactate distribution volume scaled to body mass. Yet adipose tissue neither contributes to glycolytic energy production nor participates meaningfully in lactate distribution. Scaling to fat-free mass has been shown to improve the correspondence between calculated glycolytic energy and measured sprint work in cyclists (Meixner et al. 2024), with similar work–lactate relationships observed across sexes when fat-free mass is used as the scaling denominator (Meixner et al. 2026). In athletes with higher body fat fractions, body-mass-scaled LAO₂ will therefore underestimate glycolytic rate per unit of metabolically active tissue. Fat-free mass is the more appropriate denominator in heterogeneous or higher-body-fat cohorts, although in the present low-fat sample the choice of denominator did not affect the results (see Results). However, a validated fat-free-mass-based LAO₂ equivalent for use in energy-share modeling is currently lacking, and its derivation represents an important avenue for future work.

The regression models are exploratory and hypothesis-generating: with *n* = 17 and up to three predictors (≈ 6 observations per predictor) they fall below the commonly recommended 10–15 observations per predictor, so the coefficients are prone to overfitting and the resulting equations should be interpreted with caution and validated in independent samples before applied use. The fixed test order (12 s before 60 s) may introduce order effects, although the prolonged individualized recovery and return to baseline concentrations mitigate this concern. Data were pooled across sexes due to insufficient female sample size (*n* = 3), limiting sex-specific inference.

Generalizability of the present findings is currently limited to continuous, cyclic locomotion tasks such as sprint cycling. In intermittent sports with pronounced load-unload phases (e.g., rowing, skating), altered metabolic recovery dynamics may shift optimal assessment windows toward longer durations, warranting modality-specific validation. Future studies should examine transferability across sex, training status, and exercise modality and aim to derive simplified surrogate models that preserve physiological validity while improving feasibility for routine diagnostics.

## Conclusion

Maximal lactate accumulation rate can be estimated with physiological consistency from a single maximal sprint using a time-resolved energy-share approach with dynamic efficiency modeling. Peak glycolytic activation occurred before the conventional 12 s endpoint (group mean near 9 s), consistent with systematic underestimation by fixed-duration protocols. The magnitude of vLamax was associated with maximal isometric force and relative aerobic capacity, reflecting the balance between glycolytic production and oxidative pyruvate disposal. The mechanically derived alactic phase duration (tFF) was supported as a measurement-based alternative to model-dependent talac estimation. These findings support shorter, physiologically grounded sprint diagnostics, with approximately 10 s representing a practical compromise between validity and feasibility, and provide a framework for individualized metabolic assessment in high-performance sport. Whether the reconstructed peak is the appropriate input for integrative metabolic models, whose constants were calibrated against gold-standard estimates, is an open question; answering it would require joint re-estimation against independent data.

## Abbreviations

Abbreviation: Definition
ADP: Adenosine diphosphate
ATP: Adenosine triphosphate
BLCmax: Maximal blood lactate concentration
BM: Body mass
CE: Caloric equivalent
EPOC: Excess post-exercise oxygen consumption
FFM: Fat-free mass
Fmax: Maximal (isometric) pedal force
F–v: Force–velocity (profile)
GS: Gold standard
Glymax: Maximal glycolytic flux (corrected for oxidative pyruvate disposal)
IA: Indirect assessment
LAO₂: Oxygen–lactate equivalent
LoA: Limits of agreement
LOO-CV: Leave-one-out cross-validation
NIRS: Near-infrared spectroscopy
PCr: Phosphocreatine
Pi: Inorganic phosphate
Pmax: Maximal power
PRopt: Optimal pedalling rate
RER: Respiratory exchange ratio
RMSE: Root mean square error
SEE: Standard error of estimate
SmO₂: Muscle oxygen saturation
STROBE: STrengthening the Reporting of OBservational studies in Epidemiology
talac: Alactic phase duration
tFF: Time of first deviation from the fatigue-free force–velocity profile
tvLamax: Time to peak lactate accumulation rate
V̇CO₂: Carbon dioxide output
V̇O₂: Oxygen uptake
V̇O₂peak: Peak oxygen uptake
V̇O₂peak,rel: Body-mass-normalised peak oxygen uptake
vLa(t): Instantaneous (time-resolved) lactate accumulation rate
vLamax: Maximal lactate accumulation rate (vLamax, IA indirect; vLamax, GS gold standard)
WAER(t): Aerobic (oxidative) energy
WBLC(t): Glycolytic energy (lactate-associated)
WPCr(t): Phosphocreatine-derived (alactic) energy
WTOT(t): Total metabolic energy turnover
ΔBLC: Net increase in blood lactate concentration
η(t): Dynamic gross efficiency
τPCr: Time constant of phosphocreatine dephosphorylation
τSmO₂: Time constant of SmO₂ recovery
τW: Time constant of total metabolic energy turnover
